# Evaluation of IL-23p19/Ebi3 (IL-39) gingival crevicular fluid levels in periodontal health, gingivitis, and periodontitis

**DOI:** 10.1007/s00784-022-04681-w

**Published:** 2022-08-20

**Authors:** Aysegul Sari, Serdar Dogan, Luigi Nibali, Serhat Koseoglu

**Affiliations:** 1 Department of Periodontology, Faculty of Dentistry, Hatay Mustafa Kemal University, Hatay, Turkey; 2 Periodontology Unit, Centre for Host-Microbiome Interactions, Faculty of Dentistry, Oral & Craniofacial Sciences, King’s College London, London, UK; 3 Department of Medical Biochemistry, Faculty of Medicine, Hatay Mustafa Kemal University, Hatay, Turkey; 4 Department of Periodontology, Faculty of Dentistry, Istanbul Medeniyet University, Istanbul, Turkey

**Keywords:** Periodontal disease, Periodontitis, Inflammation, IL-23p19/Ebi3 (IL-39), Cytokine

## Abstract

**Objectives:**

IL-23p19/Ebi3 (IL-39) was described as a new IL-12 family member. The aim of this study is to evaluate the gingival crevicular fluid (GCF) IL-39 levels in periodontal diseases and health and to correlate them to GCF levels of IL-1β and periostin.

**Materials and methods:**

Sixty-six adult patients were included in the study. The study design was comprised of three groups, each containing 22 individuals: the periodontally healthy (PH), gingivitis (G), and periodontitis (P) groups. The clinical periodontal parameters were recorded and GCF samples were collected from the participants. GCF interleukin (IL)-39, IL-1β, and periostin levels were examined using the enzyme-linked immunosorbent assay.

**Results:**

GCF IL‑1β, periostin, and IL-39 levels were higher in the P and G groups than in the PH group (*p* < 0.001). Positive correlations were detected between all GCF biochemical parameters and clinical periodontal parameters (*p* < 0.05). In the multivariate generalized linear regression analysis, the P (β = 37.6, 95% CI = 22.9–52.4) and G (β = 28.4, 95% CI = 15.8–41) groups were associated with GCF IL-39 levels (*p* < 0.001).

**Conclusion:**

IL-39 levels were elevated in the presence of periodontal disease paralleling the increase in IL‑1β and periostin levels. IL-39 may have a role in the periodontal inflammation process.

**Statement of clinical relevance:**

IL-39, a new cytokine from the IL-12 family, can be a possible predictor marker of periodontal diseases.

**Supplementary Information:**

The online version contains supplementary material available at 10.1007/s00784-022-04681-w.

## Introduction

Periodontal diseases are multifactorial chronic inflammatory diseases. The main driver of periodontal disease in susceptible individuals is the interaction and development of dysbiotic communities and destructive inflammation which co-develop and are reciprocally reinforced [1]. The nature of microbial stimuli that trigger disruptions in periodontal homeostasis are determinants of host responses in the gingiva [2]. Host modulation therapies, such as anti-cytokine therapy and engineered exosomes derived from dendritic cells, can restore the balance between pro-inflammatory and anti-inflammatory mediators and create an environment that can reverse dysbiosis [3, 4]. For these reasons and to understand the role of cytokines in periodontitis, cytokine studies are currently being conducted.

The complex cytokine network involved in the immune system includes specific cytokine receptors, pro-inflammatory and anti-inflammatory cytokines. In this context, cytokines in inflamed periodontal tissues are thought to have a prominent effect on the host modulation and initiation and progression of periodontal disease [5]. One of the most studied is interleukin 1 (IL-1), which is an important proinflammatory mediator in the host inflammatory response. IL-1β has a crucial role in activating proteinase, collagenase, stimulating the production of prostaglandin E2, and enhancing bone resorption and degradation of the extracellular matrix in periodontal diseases [6, 7].

Periodontal tissue destruction is mediated by interactive relationships between proinflammatory and anti-inflammatory mediators in the host response [8]. Periostin, a secreted hematopoietic stem cell protein of 90 kDa containing glutamate, is expressed in the periosteum and periodontal ligaments [9]. It is thought to enhance cell survival and differentiation, promote cell adhesion and fibrogenesis, and to affect periodontal tissue remodeling and bone formation by affecting differentiation, adhesion, and proliferation of osteoblasts [10, 11]. This protein regulates cell functions to favor tissue regeneration by different signaling pathways such as the PI3K/Akt/mTOR, αvβ3 integrin/FAK/PI3K/Akt, αvβ3 integrin/extracellular-related kinase [10, 12–14].

Previous studies suggested that periostin expression is regulated by various factors such as interleukin-4, interleukin-13, and transforming growth factor-β (TGF-β) [15]. Periostin expression in the periodontal ligament is regulated by TGF-β, mechanical stress such as during mastication and tooth movement [11]. A study stated that periostin values increased in the presence of periodontitis in gingival crevicular fluid (GCF) [16].

The IL-12 family of cytokines regulates adaptive immune responses and endogenous responses by inducing natural killer and Th1 cells to produce interferon-gamma (IFN-γ) [17]. IL-1β has a major role in IL-12β b production and IL-12 αβ heterodimer secretion and can synergize with IFN-γ secretions of high amounts of IL-12 [18]. IL-12 is higher in GCF in the presence of periodontitis [19]. In recent years, IL-23p19/Ebi3 (IL-39) was described as a new IL-12 family member as an additional combination to the four known members of this cytokine by Wang et al. [20]. This cytokine has been proposed as pro-inflammatory and a potential predictor and prognostic marker in acute coronary syndrome (ACS) [21]. The association of IL-39 with inflammatory diseases, except for ACS and systemic lupus erythematosus, has not been clarified yet [22].

To our knowledge, there is no study in the literature investigating IL-39 cytokine levels in periodontal disease. We aimed to assess IL-39 levels in GCF in patients with different periodontal phenotypes and to correlate them with IL-1β and periostin levels. Our null hypothesis is that no differences exist in GCF levels of patients with periodontitis, gingivitis, and gingival health.

## Materials and methods

The present case–control study was conducted in the Department of Periodontology in the Faculty of Dentistry at Hatay Mustafa Kemal University, Hatay, Turkey. The study protocol was approved by the Ethics Committee for the Use of Human Subjects in Research of Hatay Mustafa Kemal University (Protocol No: 2021/70) and the study was carried out in accordance with the tenets of the Declaration of Helsinki. Individuals were included in the study from July 2021 through November 2021. Written informed consent was obtained from each participant before the clinical periodontal examination.

### Study groups

Sixty-six adult individuals were enrolled in the study. Participants were recruited into 3 sex, age-, and body mass index (BMI)-matched groups with 22 participants in each periodontally healthy (PH), gingivitis (G), and periodontitis (P) groups. In accordance with the demographic variables of the patients included in the P group, the patients in the G and PH groups were included.

The inclusion criteria included the following: (a) individuals who had more than 18 teeth; (b) individuals who had had no antibiotic therapy in the past 3 months; (c) individuals who had no history of periodontal treatment in the past 6 months; and (d) individuals who had never smoked.

The exclusion criteria included the following: (a) individuals who had any self-reported systemic condition or disease, which are confounding factors, e.g., AIDS, rheumatoid arthritis, cardiovascular diseases, diabetes; (b) individuals were current or former smokers; (c) individuals were pregnant, and (d) individuals with BMIs ≥ 25 kg/m^2^.

### Clinical measurements

#### Periodontal parameters

Periodontal clinical parameters were recorded by a single calibrated examiner (k = 0.93) (author AS). Intra-examiner agreement was determined for CAL. The intra-examiner reproducibility was determined through repeated examinations of 10 subjects with a one-hour interval. Clinical periodontal measurements were assessed using the following periodontal measurements for periodontal diagnosis.

The measurements were performed using a Williams periodontal probe (Hu-Friedy, Chicago, IL, USA) and included probing pocket depth (PPD), clinical attachment level (CAL), plaque index (PI) [23], gingival index (GI) [24], and percentage bleeding on probing (BOP) [25] at six sites per tooth (mesio-buccal, buccal, disto-buccal, mesio-lingual, lingual and disto-lingual) on each tooth.

Diagnosis of periodontal diseases and conditions was made according to the radiographic and clinical diagnostic criteria proposed by the 2017 World Workshop on Classification of Periodontal and Peri-implant Diseases and Conditions [26]. Individuals with a BOP < 10% without attachment loss and radiographic bone loss were considered to have periodontal health [27]. Only generalized gingivitis patients were included in this study. Individuals presenting with a BOP ≥ 30%, and PPD ≤ 3 mm without radiographic bone loss and attachment loss were considered to have gingivitis [28]. The criteria for periodontitis included patients with CAL ≥ 5 mm in two or more interproximal sites and PPD ≥ 6 mm in one or more interproximal sites. Only stage III–IV (severe) periodontitis was included in the present study [29].

#### Collection of GCF samples

GCF samples were collected following 8 h of night fasting and one week after clinical periodontal measurements [30]. The samples were collected from the teeth side with BOP positive in patients with gingivitis and CAL ≥ 5 mm, PPDs ≥ 5 mm, and BOP positive in patients with periodontitis. Six GCF samples were taken per participant. Samples were collected from a mesio-buccal and a disto-palatinal site on each of three teeth (incisors, premolars, and molars). Saliva contamination was prevented by isolation with cotton rolls and gently air-drying the sampling area. Samples contaminated with saliva or blood were not included. Plaque was gently removed from the sampling area by the periodontal curette. The samples were collected within 30 s with standardized paper strips (Periopaper; Oraflow Inc., Plainview, NY) by the orifice method [31]. The volumes were measured on a pre-calibrated electronic gingival fluid measuring device (Oraflow Inc., Plainview, NY)^||^ [32]. The values of the electronic device were referenced to a standard curve and converted to an actual volume (µl). All of the Periopaper strips were pooled in plastic Eppendorf microcentrifuge tubes. They were stored at – 80 °C until the biochemical analysis.

At a later stage, samples were thawed, 750 μl phosphate buffer (Phosphate Buffered Saline, pH: 7.00, 137 mM NaCl, 10 mM Na_2_PO_4_, and 2.7 mM KCl) was added into the Eppendorf microcentrifuge tubes containing the sample strips and samples were eluted for 30 min at room temperature before the assay [33]. The tubes were centrifuged at 12,000 × *g* for 15 min. After removing the strips, the supernatants were used for the measurement of IL-1β, periostin, and IL-39.

### Laboratory analyses

#### Measurement of IL‑1β levels in GCF samples

IL-1β levels were assayed with commercially available kits using the enzyme-linked immunosorbent assay (ELISA) method (Elabscience, catalog no: E-EL-H0149). The optical density was measured spectrophotometrically at a wavelength of 450 nm (Thermo Scientific MultiscanGo, Finland). The assay ranges for the IL-1β kit were 7.81–500 pg/mL, sensitivity 4.69 pg/mL, and the intra- and interassay coefficients of variance (CV%) were < 10%. The results were presented as pg.

#### Measurement of periostin levels in GCF samples

Periostin levels were determined with commercially available kits using the ELISA method (Elabscience, catalog no: E-EL-H6113). The optical density was measured spectrophotometrically at a wavelength of 450 nm (Thermo Scientific MultiscanGo, Finland). The assay ranges for the periostin kit were 3.13–200 ng/mL, sensitivity 1.88 ng/mL, and the intra- and interassay coefficients of variance (CV%) were < 10%. The results were presented as pg.

#### Measurement of IL‑39 levels in GCF samples

IL-39 levels were studied with commercially available kits using the ELISA method (MyBioSource, catalog no: MBS167915). The optical density was measured spectrophotometrically at a wavelength of 450 nm (Thermo Scientific MultiscanGo, Finland). The assay ranges for the IL-39 kit were 2–600 ng/L, sensitivity 1.07 ng/L, and the intra- and interassay coefficients of variance (CV%) were < 10%. The results were presented as ng.

### Statistical analysis

The main study outcome was IL-39 (pg/30sn) GCF levels between different periodontal phenotypes (periodontitis, gingivitis, healthy). In the absence of previous data about IL-39 GCF levels, a convenience sample of 66 participants was chosen for this study. Post-hoc sample size calculation revealed that 22 patients per group would give 100% power for effect size f of 1.04 (standard deviation: 13.72) and α = 0.05. Gpower package version 3.1 was used for sample size calculations.

The normality of continuous variables was evaluated by Shapiro–Wilk’s test. Non-parametric statistical methods were performed for values with skewed distribution. Descriptive statistics were presented as median (interquartile range) for the non-normally distributed variables.

Kruskal–Wallis test was performed for comparison of more than two non-normally distributed variables and Dunn multiple comparison test was performed for post hoc pairwise multiple comparison analyses. One-way ANOVA test was performed for comparison of more than two normally distributed groups and Tukey test was performed for post hoc pairwise multiple comparison analyses. In addition, significance values have been adjusted by the Bonferroni correction for multiple tests. The chi-square test was used to analyze the associations between categorical variables. ROC analysis was performed in order to evaluate diagnostic performance of IL-39 levels (pg/30sn) for periodontal diseases. Area under curve value and also sensitivity and specificity values have been calculated according to presence of periodontal diseases and absence. The correlation between two non-normally distributed variables was evaluated by Spearman Rho correlation coefficient. Multivariate generalized linear model was performed to evaluate the association between the presence of periodontal disease and IL-39 levels. The variables were included in the multivariate model, provided that significance at the 5% level was obtained in the univariate generalized linear model analysis. Variance inflation factors were calculated to check multicollinearity. IL-39 levels (pg/30sn) were selected as dependent variable, while age, sex, BMI, IL-1β levels (pg/30sn), periostin levels (pg/30sn), and periodontal groups were set as independent variables in the multivariate model. The MedCalc Statistical Software (ver. 12.7.7; MedCalc Software bvba, Ostend, Belgium) was used for statistical analyses and *p* < 0.05 was considered statistically significant.

## Results

### Demographic findings

Table 1 shows the demographic characteristics of the groups. Sex, age, and BMI were not statistically significantly different among the groups (*p* = 0.999, *p* = 1, and *p* = 0.997 respectively).

**Table 1:** The demographic characteristics of the groups

Variable	PH group (*n* = 20)	G group (*n* = 20)	P group (*n* = 20)	*p* value*
Age (IQR: 25–75)	33 (29.0–45.3)	32.5 (25.8–51.3)	39.5 (29.5–46)	0.999
Sex (males/females) *n* (%)	9/13	9/13	9/13	1
BMI (kg/m^2^) (IQR: 25–75)	23.2 (21.4–245)	23.2 (21.4–24.5)	24 (19.6–24.7)	0.997

^*^ *P* values obtained from Kruskal Wallis test and Chi-square test

Data are expressed as median and 25% to 75% and *n* (%). Statistically significant at *p* < 0.05

*PH* periodontally healthy, *G* gingivitis, *P* periodontitis, *BMI* body mass index

### Clinical findings

Table 2 shows the clinical periodontal parameters. PI, GI, BOP (%), and GCF volume were higher in the P and G groups than in the PH group (*p* < 0.001). As by definition, PPD was higher in the P and G groups than in the PH group and was higher in the P group than in the G group (*p* < 0.001). CAL were higher in the P group than in the G and PH groups (*p* < 0.001).

**Table 2:** Clinical periodontal parameters and GCF volume among all groups

Variable	PH group (*n* = 22)	G group (*n* = 22)	_P group (*n* = 22)_	*p* value
PI	0.05 (0–0.5)	1.8 (1–2.1) †	2.6 (2.0–3.0**) †**	< 0.001*
GI	0.2 (0.05–0.3)	2 (1.9–2.2) †	2.74 (2–2.89) †	< 0.001*
BOP (%)	3.1 (0–7.1)	90.63 (83.37–100) †	96.43 (81.25–100) †	< 0.001*
PPD	1.5 ± 0.2	3.2 ± 0.5^**†**^	4.2 ± 0.7^**†,** ‡^	< 0.001**
CAL	0 (0–0)	0 (0–0)	4.5 (3.8–4.8) †, ‡	< 0.001*
Number of missing teeth	0 (0–1)	1 (0–4) †	2 (0–5.5) †	0.004*
GCF volume (μl)	0.1 (0.1–0.2)	0.6 (0.4–0.8)†	0.8 (0.4–1.1) †	< 0.001*

^*^ *P* values obtained from Kruskal–Wallis test and Dunn multiple comparison test for non-parametric variables

^**^ *P* values obtained from Anova test and Tukey test for parametric variables

Significance values adjusted by the Bonferroni correction for multiple tests

Data are expressed as median and 25% to 75% and mean ± SD

Statistically significant at *p* < 0.05; † *p* < 0.05 versus PH; ^‡^ *p* < 0.05 versus G

*PH* periodontally healthy, *G* gingivitis, *P* periodontitis, *PI* plaque index, *GI* gingival index, *BOP* percentage bleeding on probing, *PPD* probing pocket depth, *CAL* clinical attachment level, *GCF* gingival cervicular fluid

### Laboratory findings

Table 3 shows the intergroup comparisons of biochemical markers. GCF IL‑1β, periostin, and IL-39 total amounts were higher in the P and G groups than in the PH group (*p* < 0.001). GCF IL‑1β and perostin concentrations were not significantly different among the groups (*p* = 0.571 and *p* = 0.071 respectively). GCF IL-39 concentrations were higher in the PH group than in the P and G groups (*p* < 0.001).

**Table 3:** Biochemical markers among all groups

Variable	PH group (*n* = 22)	G group (*n* = 22)	_P group_ (*n* = 22)	*p* value
IL‑1β total amount (pg/30sn)	18 ± 6.4	68.5 ± 26.2 †	86 ± 29.7 †	< 0.001*
IL‑1β concentration (pg/μl)	24.55 (14.6–32.1)	19.2 (12.8–25.6)	17.8 (12.6–27.3)	0.571
Periostin total amount (ng/30sn)	19.3 ± 9.5	59.5 ± 24.2 †	70.5 ± 29.1 †	< 0.001**
Periostin concentration (ng/μl)	23.2 (14.8–39.2)	15.4 (11.7–20.1)	13.9 (11.4–19.5)	0.071
IL-39 total amount (pg/30sn)	102.9 (92.6–113.3)	123.8 (119.5–130.1) †	133 (127–139) †	< 0.001*
IL-39 concentration (pg/μl)	121.1 (73.65–223.9)	34.6 (27.6–51.7) †	26.8 (21.7–53.9) †	< 0.001*

^*^ *P* values obtained from Kruskal–Wallis test and Dunn multiple comparison test for non-parametric variables

^**^ *P* values obtained from Anova test and Tukey test for parametric variables

Significance values adjusted by the Bonferroni correction for multiple tests

Data are expressed as median and 25% to 75% and mean ± SD

Statistically significant at *p* < 0.05; †*p* < 0.05 versus PH

*PH* periodontally healthy, *G* gingivitis, *P* periodontitis, *IL*‑*1β* interleukin 1β, *IL*-*39* interleukin 39

### Correlations

Table 4 shows correlations between clinical periodontal parameters and GCF IL‑1β, periostin, and IL-39 total amounts. Positive correlations were detected between GCF IL‑1β, periostin, and GCF IL-39 total amounts and all clinical periodontal parameters (*p* < 0.05).

**Table 4:** Correlations between clinical periodontal parameters and total amount of cytokines

Variable*	IL‑1β total amount (pg/30sn)	Periostin total amount (ng/30sn)	IL-39 total amount (pg/30sn)
PI	*r* = 0.581 *p* ≤ 0.001	*r* = 0.579 *p* ≤ 0.001	*r* = 0.640 *p* ≤ 0.001
GI	*r* = 0.593 *p* ≤ 0.001	*r* = 0.579 *p* ≤ 0.001	*r* = 0.621 *p* ≤ 0.001
BOP (%)	r = 0.586 *p* ≤ 0.001	*r* = 0.606 *p* ≤ 0.001	*r* = 0.521 *p* ≤ 0.001
PPD	*r* = 0.707 *p* ≤ 0.001	*r* = 0.657 *p* ≤ 0.001	*r* = 0.684 *p* ≤ 0.001
CAL	*r* = 0.709 *p* ≤ 0.001	*r* = 0.657 *p* ≤ 0.001	*r* = 0.701 *p* ≤ 0.001
Number of missing teeth	*r* = 0.363 *p* = 0.003	*r* = 0.247 ***p*** = 0.046	*r* = 0.348 *p* = 0.004

Spearman’s rank correlation coefficient

Statistically significant at *p* < 0.05

*PI* plaque index, *GI* gingival index, *BOP* percentage bleeding on probing, *PPD* probing pocket depth, *CAL* clinical attachment level, *IL*‑*1β* Interleukin 1β, *IL*-*39* interleukin 3

Table 5 shows univariate and multivariate generalized linear regression analyses of factors affecting GCF IL-39 total amounts. Age, sex, and BMI were not associated with GCF IL-39 total amounts (*p* > 0.05). P (β = 33.6, 95% CI = 42.6–24.6) and G (β = 25.3, 95% CI = 16.3–34.3) groups, Periostin total amounts (β = 0.29, 95% CI = 0.14–0.44), and IL‑1β total amounts (β = 0.29, 95% CI = 0.17–0.4) were associated with GCF IL-39 total amounts (*p* < 0.001) in the univariate generalized linear regression analysis. P (β = 37.6, 95% CI =  − 22.9–52.4) and G (β = 28.4, 95% CI = 15.8–41) groups were associated with higher GCF IL-39 total amounts (*p* < 0.001 and 0.031, respectively) in the multivariate generalized linear regression analysis.

**Table 5:** Generalized linear regression analysis of factors affecting IL-39 total amount cytokine levels

		Univariate generalized linear model		Multivariate generalized linear model	
		Coefficient (95% CI)	*p* value	Coefficient (95% CI)	*p* value^†^
Groups	P	33.6 (24.6–42.6)	< 0.001	37.6 (− 22.9–52.4)	< 0.001
	G	25.3 (16.3–34.3)	< 0.001	28.4 (15.8–41)	< 0.001
	PH	1 (Reference)	N/A	1 (Reference)	N/A
	Age	0.050 (− 0.41–0.51)	0.829	–	**–**
	BMI	1.45 (− 0.77–3.71)	0.199	–	**–**
	Sex Female	− 5.2 (− 15.5–4.8)	0.305	–	**–**
	Periostin	0.29 (0.14–0.44)	< 0.001	0.057 (− 0.24–0.13)	0.543
	IL-1β	0.29 (0.17–0.4)	< 0.001	0.16 (− 0.19–0.16)	0.861

Statistically significant at *p* < 0.05

*CI* confidence interval, *N*/*A* not applicable, *PH* periodontally healthy, *G* gingivitis, *P* periodontitis, *BMI* body mass index

### Roc analysis

**Fig. 1:**
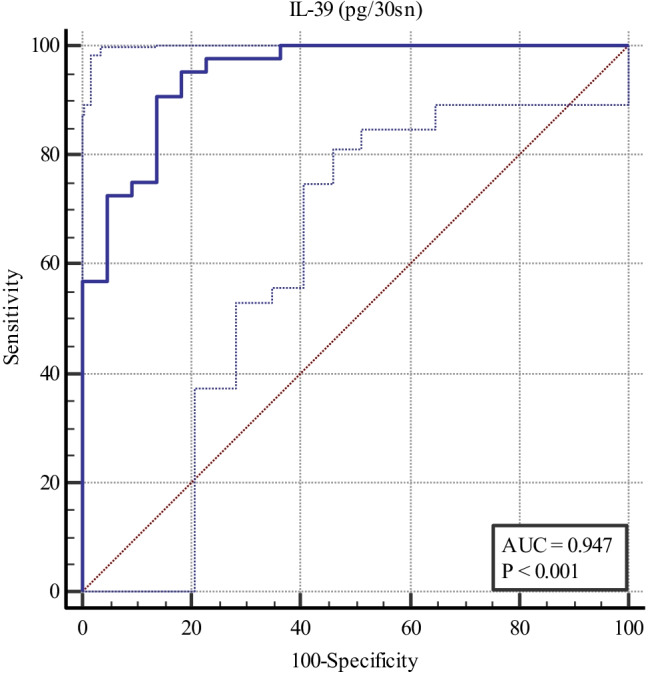
The graphic of IL-39 (pg/30sn) sensivity and specificity for periodontal diseases

In ROC analysis, area under curve (AUC) value was calculated as 0.947 for IL-39 levels (pg/30sn). Sensitivity was calculated as 95.5%, whereas specificity values as 81.82%. The cut off value was found as > 114.7 (Fig. 1).

## Discussion

This is the first study to evaluate GCF IL-39, IL-1β, and periostin levels in periodontitis, gingivitis and periodontal health to the best of the authors’ knowledge. The findings of the present study show that IL-39, IL-1β, and periostin levels were elevated in GCF in presence of periodontal disease.

We presented data for both total levels and concentrations and discussed both in the current study. It is interesting to notice that higher ‘concentrations’ of the 3 studied cytokines were detected in healthy vs. gingivitis and periodontitis patients. However, total amounts of cytokines in GCF samples per sampling time have been suggested as a better indicator of relative GCF constituent activity rather than concentration because concentrations are directly affected by the sample volume [34–36]. Several studies have indicated that the expression of GCF data as the total amount per standardized sampling time is a more sensitive way than reporting them as concentration, and should be used when estimating periodontal disease activity [34, 35, 37, 38]. This is particularly true in the present study, as GCF volume was 8 times higher in periodontitis vs. controls and 6 times higher in gingivitis vs. controls. Therefore, total amounts have been taken as the main results in the current study.

The main novel finding of the study relates to IL-39, which was discovered as a new inflammatory marker in recent years, is produced in vivo and in vitro by activated mouse B cells and upregulates neutrophils [20, 39]. IL-39 total levels were higher in GCF of periodontitis and gingivitis patients compared with healthy controls. Also, P and G groups were associated with higher GCF IL-39 total levels compared to PH group in generalized linear regression analysis. These results were independent confounding factors such as BMI, age, and sex. Therefore, IL-39 levels appeared to increase in the presence of periodontal infection, suggesting that IL-39 may be an inflammatory marker in humans, playing a role in the inflammatory processes of periodontal diseases as well as in different disease groups. This is in agreement with some previous literature [21, 39, 40]. In a preclinical study, Wang et al. suggested that IL-39 may have a role in lupus immunopathogenicity [20]. It has been demonstrated that the number of B cells involved in the production of IL-39 increases in mice with lupus, and the severity of the disease decreases as a result of suppression of IL-39 [39]. Also, it was noticed that anti-IL-39 antibodies have a therapeutic effect in a lupus-like pathology in mice, suggesting that IL-39 could be a potential marker for the treatment of systemic lupus erythematosus due to its proinflammatory nature [41]. Another study showed that IL-39 affects pancreatic cancer by pro-tumorigenic function [42]. In a clinical study, it has been reported that IL-39 levels in serum are increased in patients with ACS compared with healthy individuals and there were positive correlations between IL-39 levels and hs-CRP [21]. In addition, it has been shown that serum IL-39 levels were higher in patients with relapsing–remitting multiple sclerosis and neuromyelitis optica spectrum disorders than healthy controls [43]. In contrast, a few studies failed to show evidence of IL-39 production by human cells. Two in-vivo studies noticed that, although IL-39 is secreted in human cells, it may be below the detection limit or it has no functional response [40, 44]. In these studies, the choice of cells was made according to previous studies that were carried out on mice [40, 44]. The discrepancy between the results of these studies and those of the present study may be due to the production of IL-39 by other cells or targeting different cells in humans as opposed to mice. Another reason for this discrepancy can be that several inducing pathways interact in in-vivo studies as opposed to one-way interactions in in-vitro studies.

Further evidence of a potential pro-inflammatory nature of IL-39 derives from its correlation with IL-1β levels in the present study. IL-1β is a key cytokine in periodontal diseases because of its dual function in collagen degradation and effects on enhancing bone resorption and inhibiting bone formation [6, 45, 46]. GCF IL‑1β total amounts were higher in the P and G groups than in the PH group. Compatible with the current study results, previous studies have demonstrated that IL-1β is usually found in higher levels in patients with periodontitis than in the healthy controls in GCF and different tissue samples [47, 48] including saliva [49], although its potential use as a biomarker is yet unclear due to inconsistencies across studies [50]. The association between IL-39 and IL-1β levels in generalized linear regression analysis is supported by a study which demonstrated that IL-1 plays a role through upregulation of IL-12 secretion by dendritic cells in innate and adaptive immunity [18]. Another study noticed that IL-12 needs the induction of IL-1 beta to stimulate IFN-gamma production by natural killer cells and IL-1 plays a role in the IL-12-mediated inflammatory response to some bacteria [51]. The results of these previous studies are consistent with the fact that IL-39, a cytokine from the IL-12 family, correlates with IL-1 levels in the presence of periodontal inflammation.

Periostin, playing a role in the remodeling of periodontal tissues, is an important adhesion molecule and structural mediator [10]. This protein is localized among the cytoplasmic processes of periodontal cementoblasts and fibroblasts and the adjacent collagen fibrils [52]. Thus, it can be used as a marker of the periodontal regeneration process [53]. The results of the present study indicated that periostin total amounts were higher in the P and G groups than in the PH group. There are some contradictory results in the literature. It has been noticed that *Porphyromonas gingivalis* lipopolysaccharide and TNF-α decrease periostin levels in the periodontal ligament [54]. In contrast with the present study, GCF periostin levels were previously shown to decrease in relation to the severity of periodontitis [55, 56]. These results may support the idea that periodontal inflammation can cause of periostin downregulation. However, in agreement with the present study, Arslan and co-workers found that periostin GCF levels decreased from the periodontitis group to the gingivitis and to the healthy control group [16]. As in the present study, samples had been taken from bleeding sites (GI = 2 and BOP positive). It can be considered that the inconsistency with some of the previous studies may be related to the differences in the disease activity in the site of the GCF samples taken. Previous studies have also shown that periostin differentially expressed and upregulated contributes to the development and progression of various inflammatory diseases such as cancer, diabetes, and bowel disease [57, 58]. The results may suggest that in addition to playing a role in the regeneration processes, periostin can also be effective in regulating inflammatory responses [11, 59]. It can be thought that periostin increases as a protective mechanism and response in the periodontal inflammation process. In this process, periostin may increase to allow tissue repair and remodeling.

Lending further support to the association with the periodontal groups in this study are the correlations between the assessed biomarkers and continuous measures of disease severity. We detected positive correlations between GCF IL‑1β, periostin and IL-39 levels, and all periodontal clinical parameters, in keeping with some previous studies [21, 43, 60, 61]. In the Roc analysis, IL-39 showed strong performance in presence of periodontal disease. IL-39 showed a high specificity for periodontal disease and high sensitivity for periodontal health. Thus, GCF IL-39 levels could potentially be used as a biomarker for the presence of periodontal diseases, upon confirmation of the findings of the present study.

The strengths of this study are the novelty in assessing GCF levels of IL-39, the rigorous pre-sampling protocol, and the fact that smokers were excluded, while sex, age, and BMI were matched among the groups to reduce the risk of finding spurious associations [62, 63]. Biochemical findings are given as both concentration and total amount values in the present study.

The case–control design and relatively small sample size may be limitations of the present study.

## Conclusions

The present study findings showed that GCF IL-39, IL‑1β, and periostin total amounts were higher in patients with periodontitis and gingivitis than in the healthy controls. These results suggest that the presence of periodontal diseases is associated with increased GCF IL-39, IL-1β, and periostin levels. IL-39, a new cytokine from the IL-12 family, may have a role in the periodontal inflammation process and it can be a possible marker of periodontal diseases. Further studies with larger sample size and longitudinal assessment post-treatment are needed to clarify the functions of IL-39 and its possible role in the pathogenesis of periodontal diseases.

## Supplementary Information

Below is the link to the electronic supplementary material.

Supplementary file1 (DOC 79 KB)
